# Human enteroviruses in cerebrospinal fluid of children with suspected aseptic meningitis: A study in northern Iran

**DOI:** 10.22088/cjim.8.2.112

**Published:** 2017

**Authors:** Farzin Sadeghi, Masoumeh Talebi-Nesami, Rahim Barari-Savadkouhi, Ali Bijani, Elahe Ferdosi-Shahandashti, Yousef Yahyapour

**Affiliations:** 1 Cellular and Molecular Biology Research Center, Health Research Institute, Babol University of Medical Sciences, Babol, Iran.; 2 Non-Communicable Pediatric Diseases Research Center, Health Research Institute, Babol University of Medical Sciences, Babol, Iran.; 3 Social Determinants of Health (SDH) Research Center, , Health Research Institute, Babol University of Medical Sciences, Babol, Iran.; 4 Department of Advanced Technologies in Medicine (SATiM), School of Medical Biotechnology, Tehran University of Medical Sciences, Tehran, Iran.; 5 Infectious Diseases and Tropical Medicine Research Center, Health Research Institute, Babol University of Medical Sciences, Babol, Iran.

**Keywords:** Enterovirus, Aseptic meningitis, Cerebrospinal fluid, Real-Time RT-PCR

## Abstract

**Background:**

Enterovirus (EV) infections are one of the most common causes of aseptic meningitis in pediatrics. To diagnose EV meningitis, virus isolation in cell cultures is often time consuming and lacks sensitivity to be of clinical relevance. This makes the virus culture results difficult to interpret. The rapid detection of EVs in cerebrospinal fluid (CSF) by molecular diagnostic techniques may improve the management of patients with aseptic meningitis. The purpose of the present study was to develop a more convenient and sensitive alternative technique to viral culture. The current investigation aimed to explore the prevalence of EVs in CSF of children with suspected aseptic meningitis in northern Iran, between June 2014 and March 2015 via the one-step real-time RT-PCR technique.

**Methods:**

A single center cross-sectional study was carried out on 50 children suspected with aseptic meningitis, aged 6 months to 13 years. The presence of EV RNA in CSF samples was screened by the use of qualitative one-step real-time RT-PCR.

**Results:**

Enteroviral RNA was detected in 9 (18%) subjects using the one-step real-time RT-PCR assay. There was significant difference between EV positive and negative subjects regarding mean age (P=0.023), mean lymphocyte percentage (P=0.001) and mean glucose levels in CSF (P=0.037). The disease onset data indicate that the majority of EV meningitis occurred in the summer.

**Conclusion:**

This study provides the first data on the prevalence and epidemiology of EV infections in children with suspected aseptic meningitis in northern Iran.

Enterovirus (EV) infections currently account for a considerable number of aseptic meningitis in patients of all age groups, especially in children (1). Clinical presentation of EV meningitis is often initially indistinguishable due to bacterial or fungal pathogens, resulting in unnecessary hospitalization and improper therapeutic regimen (2). Specific and rapid diagnosis of EV meningitis may therefore have a great importance for patient clinical management. To diagnose EV meningitis, a gold standard method involves virus culture in cerebrospinal fluid (CSF) specimens; however, this method is time-consuming and takes several days to be conclusive (3). Moreover, low virus titers in CSF and difficulties in isolation of certain EV serotypes make the virus culture results difficult to interpret (3). Nucleic acid amplification techniques such as reverse transcription (RT-PCR) methods have been introduced for the detection of EVs in CSF specimens as more convenient and sensitive alternative to viral culture (4, 5).

Several clinical studies have demonstrated the superiority of single tube (one-step real-time RT-PCR) over other molecular techniques in terms of sensitivity, total assay time and reducing the risk of amplification product carry-over (6-8). The rapid detection of EVs in CSF by one-step real-time RT-PCR may improve the management of patients with suspected meningitis, and reducing the duration of hospitalization. Hence, the facts reviewed above encouraged us to investigate the prevalence of EVs in CSF of children with suspected aseptic meningitis who referred to one of the children's hospital in northern Iran, between June 2014 and March 2015 using one-step real-time RT-PCR technique.

## Methods

**Patients and Samples:** This cross-sectional study was carried out on 50 children suspected with aseptic meningitis, aged 6 months to 13 years. They were admitted to Amirkola Children's Hospital, affiliated to Babol University of Medical Sciences, between June 2014 and March 2015. All patients had clinical presentations compatible with aseptic meningitis, including headache, vomiting, stimulation of meninges, fever and convulsion. Lumbar punctures were performed immediately after the onset of illness and CSF specimens were cultured routinely for common bacterial pathogens. None of the patients had received antibiotics prior to admission. Except for one sample, all of the CSF samples had negative cultures for bacteria. All CSF samples were immediately sent to the Microbiology Department of Babol University of Medical Sciences and were stored at -80°C until processed. The demographic and clinical characteristics of patients included in the current study are presented in table 1. This study was approved by the Ethics Committee of Babol University of Medical Sciences, and for all subjects, written informed consent was obtained.

**Viral RNA Extraction:** Viral RNA was extracted from 100 µl of CSF samples using the Cinna Pure RNA extraction kit (Cinnagene, Tehran, Iran) according to the manufacturer’s instructions. To rule out the possibility of contamination in RNA extraction, in parallel with the CSF samples, negative controls (sterile microcentrifuge tubes containing only reaction mixtures) were included. 

**One-Step Real-Time RT-PCR:** The presence of EV RNA in CSF samples was screened by the use of qualitative one-step real-time RT-PCR. Assay conditions were optimized on ABI 7300 real-time PCR system (Applied Biosystems, Branchburg, NJ, USA) with the primer sets and TaqMan probe speciﬁc for the conserved sequences in the 5'UTR of the enterovirus genome (6). Each reaction consisted of 5 µl of RNA extract, 12.5 μl YTA one step multiplex qRT-PCR smart mix(Yekta Tajhiz Azma, Tehran, Iran), 0.3 µM each primer and 0.2 µM dual-labeled probe in a 25 μl total reaction volume. The synthesis of cDNA was conducted at 48°C for 30 min and instantly followed by the activation of Taq DNA polymerase at 95°C for 30s. A total of 40 cycles was carried out, including a denaturation stage at 95°C for 15 s and a combined annealing-extension stage at 60°C for 1 min. Each real-time PCR run included reaction mixtures without RNA template as a negative control and RNA extracted from poliovirus oral vaccine as a positive control.

**Statistical analysis:** Statistical analysis was carried out using SPSS Version 20 software (SPSS, Chicago, IL). Statistical differences between groups were assessed by χ2-test. Normal distribution of the variables was checked using the Kolmogorov-Smirnov test. The differences between normal variables were analyzed by independent-samples t-test. The differences between nonparametric variables were compared by Mann–Whitney U test. A p-value of ≤ 0.05 was considered to be statistically significant.

## Results

Of the 50 children who were hospitalized with suspected aseptic meningitis, the age distribution was as follows: 36 (72%) children aged less than 2 years, 3 (6%) 2-5 years, 8 (16%) 6-10 years and 3 (6%) 11-13 years. Most of the study participants (50%) were under 12 months of age. The sex ratio of the study subjects was 2:1 (male 31, female 19). The clinical manifestation data indicate that 24 (48%) study participants had febrile convulsion, 9 (18%) participants had fever with headache, and 17 (34%) participants showed other symptoms of meningitis including fever with vomiting, fever with dysthymic disorder and fever with dizziness. Out of 50 CSF samples, enteroviral RNA was detected in 9 (18%) subjects by one-step real-time RT-PCR assay. It should be noted that in one patient who had positive CSF culture for bacteria *(Haemophilus influenzae)* enterovirus RNA also was detected. 

The mean age was 76.2±53.2 months for EV positive cases (5 males, 4 females) and 25.8±34.1 months for patients who tested negative for EVs (26 males, 15 females). Statistically significant difference between patients’ mean age and EV positivity was seen (P = 0.023). It is noteworthy that majority of positive results were observed in children aged 2-5 years (3 out of 3 cases). The mean lymphocyte percentage in CSF of EV positive subjects was 46.5±33.8, while the mean lymphocyte percentage was 8.9±27.4 for EV negative subjects. There was significant difference between EV positive and negative subjects regarding mean lymphocyte percentage in CSF (P = 0.001). In addition, mean glucose levels in CSF of EV positive subjects were significantly higher than those of EV negative subjects (P = 0.037). The disease onset data indicate that the majority of the EV meningitis occurred in the Summer of 2014 (1 in June, 1 in July, 1 in August, and 3 in September).

## Discussion

The current study describes the prevalence of EV infections in children hospitalized with suspected aseptic meningitis in one of the children's hospital in northern Iran (Amirkola Children's Hospital), between June 2014 and March 2015. It is crucial for the children's hospital to have a rapid test result for viral meningitis especially, EV meningitis (9). Rapid diagnosis of EV meningitis may lead to proper patient management, hospitalization rate decreases. minimized rate of antibiotic prescription and prevention of outbreaks (10, 11). Several lines of evidence suggest that molecular techniques are more sensitive, specific and rapid than viral culture in diagnosing EV infections (4, 5). The one-step real-time RT-PCR assay described in the present study could detect as few as 12 EV genome equivalents per reaction and generate results within few hours (6).

In the current study, EVs were detected by one-step real-time RT-PCR in 18% of patients hospitalized with suspected aseptic meningitis. The results of the present study are compatible with a number of reports with similar technique from various countries. Dalwai et al. performed a one-step real-time RT-PCR on 387 CSF samples from patients with suspected aseptic meningitis in Kuwait, and found EV RNA in 24% of cases (12). In addition, Verstrepen et al. conducted a single-tube real-time RT-PCR on CSF specimens from 70 patients suspected of viral meningitis in Belgium, and found EV RNA in 27.1% of subjects (6). 

Nonetheless, EV prevalence rate in the present study is considerably lower than a number of previous reports from Iran, which were performed with conventional RT-PCR assay. Rasti et al. performed a conventional RT-PCR on 57 CSF samples from patients with aseptic meningitis in Southern Iran (Khuzestan province), and found EV RNA in 59.6% of samples (13). Hosseininasab et al. conducted a conventional RT-PCR assay on CSF specimens from 65 patients with aseptic meningitis in Southern Iran (Fars province), and found EV RNA in 43.3% of samples (14). These discrepancies between EV prevalence rates may be due to poor hygiene and lack of healthy drinking water in southern parts of Iran or a higher rate of false positive results in conventional RT-PCR assay. 

Except for one patient with simultaneous *Haemophilus influenzae* and EV in the CSF, none of the patients were positive for bacterial causes of meningitis. Copresence of EV and *Haemophilus influenzae* in CSF samples of patients with meningitis was also reported by other investigators (14, 15). In the present study, majority of EV meningitis occurred in the summer and fall, which is in agreement with previously reported findings (13, 16-18).

 In conclusion, this study provides the first data on the prevalence of EV infections in children with suspected aseptic meningitis in northern Iran. Human EV seems to be one of the common causes of aseptic meningitis and rapid diagnosis of EV meningitis using one-step real-time RT-PCR on CSF samples can protect the patient from unnecessary use of antibiotics and allow early hospital discharge.
